# Preparation and Performance of Bitumen Modified by Melt-Blown Fabric of Waste Mask Based on Grey Relational and Radar Chart Analysis

**DOI:** 10.3390/polym16010153

**Published:** 2024-01-03

**Authors:** Peifeng Cheng, Chunmeng Zheng, Zhanming Zhang, Yiming Li, Kai Huang, Dezhong Yu, Yongcheng Ji

**Affiliations:** 1 School of Civil Engineering and Transportation, Northeast Forestry University, Harbin 150040, China; chengpeifeng@nefu.edu.cn (P.C.); zhengchunmeng123@nefu.edu.cn (C.Z.); 2022112218@nefu.edu.cn (K.H.); yongchengji@126.com (Y.J.); 2 Jiangsu Highway Engineering Maintenance Technology Co., Ltd., Nanjing 211106, China; zhanming974@163.com; 3 Longjian Road and Bridge Co., Ltd., Harbin 150001, China; 4 School of Civil Engineering, Zhejiang Shuren University, Hangzhou 310015, China; dezhong1984928@163.com

**Keywords:** modified bitumen, melt-blown fabric, waste masks, properties, radar chart

## Abstract

To effectively utilize waste mask materials in road engineering and minimize resource waste, the melt-blown fabric (MBF) of waste masks was utilized to modify the virgin bitumen. The preparation process of MBF-modified bitumen was investigated, and the physical and rheological properties of bitumen were measured. Subsequently, the blending mechanism during preparation and the dispersion morphology of the modifier were explored. Finally, the pavement performance of the mixture was investigated, and a radar chart analysis was performed to quantitatively assess the effects of MBF modification. Results suggested that the recommended preparation process of shear time, shear rate, and shear temperature was 170 °C, 4000 r/min, and 15 min, respectively. MBF enhanced the high-temperature stability of the binder and weakened the temperature susceptibility. The modification was primarily a physical process. No network structure and agglomeration formed in the bitumen after modification. The addition of MBF significantly improved the resistance of the asphalt mixture to a high-temperature deformation and water damage but harmed its low-temperature crack resistance. The comprehensive assessment results of 0% ($f_{1}$), 1% ($f_{2}$), 3% ($f_{3}$), and 5% ($f_{4}$) MBF to improve the properties of the mixture were in the following order: $f_{3}>f_{4}>f_{2}>f_{1}$, where the impact of 3% MBF was the most significant, followed by 5% and 1% MBF.

## 1. Introduction

Bitumen is a vital cementing material for flexible pavements that is widely used in road engineering, especially in highway applications [1,2]. However, a series of pavement defects, such as rutting and cracking, have been gradually appearing due to the increasing traffic loads in recent years [3]. Researchers have begun to explore new materials and modified bitumen that can provide superior performance to meet traffic requirements. From the perspective of sustainable pavement materials and environmental consciousness, it is extremely beneficial to consider crumb rubber or waste polymers as modifiers [4]. In terms of waste polymers, the use of plastic modifiers can not only effectively enhance the performance of bitumen, but also offer new approaches for waste plastic disposal and recycling. Al-Mousawi et al. [5] investigated the influence of nanosilica particles on the properties of bitumen modified with waste polypropylene polymer (WPP). They discovered that the WPP improved the high-temperature stability of the bitumen. Furthermore, the inclusion of nanosilica particles reduced the temperature sensitivity of WPP-modified bitumen by enhancing the hardness of the composite-modified binder. Zachariah et al. [6] discovered that polypropylene (PP)-modified asphalt mixture samples with crushed over-burnt brick waste possessed a comparable ability to resist rutting deformation and were durable against water damage when compared with the control sample. This indirectly reveals that PP could have a positive impact on the characteristics of bitumen. To improve the performance of SBR-modified bitumen, Vamegh et al. [7] selected PP as a modifier and investigated the impact of PP on the binder’s performance. Their conclusions declared that the stability of SBR/PP-modified asphalt mixture was markedly promoted and the moisture sensitivity was reduced as well, which was better or at least as good as SBS-modified bitumen.

In addition, PP is a high-quality material that has been widely used in manufacturing because of its stable physical and chemical properties as well as its low cost. For example, a medical mask is composed of a three-layer structure with non-woven fabric (NWF), melt-blown fabric (MBF), and non-woven fabric (NWF), whose basic component is a special polypropylene [8]. Recently, the global production of disposable medical masks has increased sharply worldwide. Waste masks belong to residual waste in domestic waste according to the standard of classification, which should be discarded in designated dustbins and transported to waste incineration plants for centralized disposal by special departments. However, the control of waste masks by authorities is still relatively careless. The report released by Oceans Asia reported that in 2020, at least 1.56 billion masks were flowing into the ocean all over the world [9]. The degradation time of plastic can be centuries because of the chemical structure of most plastics, which renders them resistant to many natural degradation processes. The slow degradation and recycling rates have made plastic an abundant polluter [10]. To make matters worse, smaller microplastic particles that form during the decomposition process will pollute the living environment of organisms, cause their death, and ultimately affect the entire ecosystem [11].

However, there are few studies on recycling waste mask resources and using them as modifiers in road engineering, with rare research on the performance of bitumen modified by waste masks. Some scholars [12] have studied the rheological behavior of modified bitumen obtained by adding waste masks. However, the blending mechanism of bitumen and the performance of modified asphalt mixtures are not comprehensively explored. Therefore, considering that a large number of discarded masks not only induce environmental pollution but also result in significant resource wastage, the main objective of this study was to explore the potential use of the MBF of waste masks as a modifying agent and provide valuable insights into the application of waste masks in road engineering. In this article, the preparation of MBF-modified bitumen was investigated by orthogonal experimental design and grey relational analysis. Then, the physical and rheological properties of modified bitumen were analyzed. The Fourier transform infrared spectroscopy (FTIR) test and fluorescence microscopy (FM) test were performed to explore the mechanism of blending in preparation, appraise the phase structure, and reflect the morphology and homogeneity of the bitumen. Meanwhile, the ability of asphalt mixtures to resist the rutting deformation, low-temperature cracking, and water damage was studied. The radar chart analysis was performed to quantitatively assess the effects of MBF on the performance of the mixture.

## 2. Materials and Methods

### 2.1. Materials

The virgin bitumen used in this research was AH-90, obtained from Suihua, Heilongjiang, China. The physical properties of AH-90 were determined. See Table 1.

The melt-blown fabric is expressed as MBF in this article. The thermal properties, molecular weight, and structural analysis of the original polymer PP from which MBF is mainly made are shown in Table 2.

**Figure 1:**
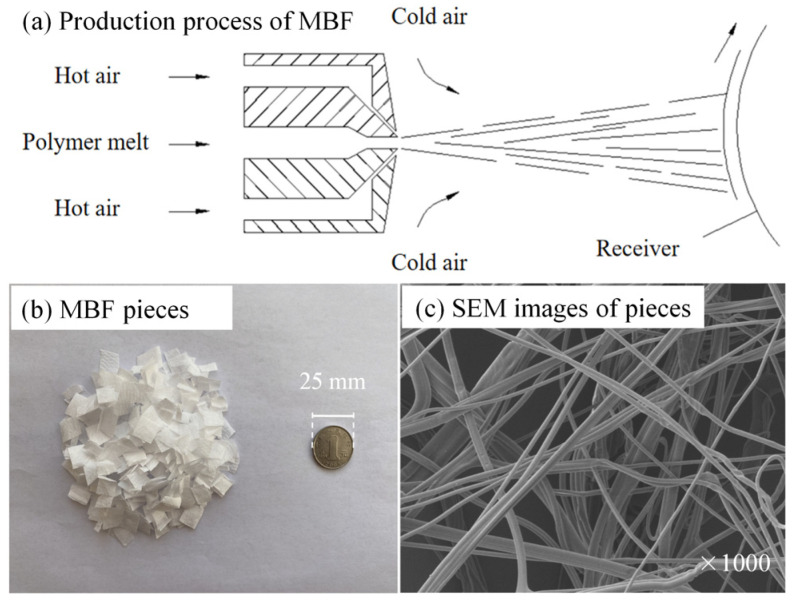
(**a**) Production process of MBF; (**b**) MBF pieces; (**c**) SEM images of pieces.

The melt-blown method is a continuous filament stretching process that was first discovered by the Naval Postgraduate School in 1954 while studying the air-jet spinning process [19]. Subsequently, the method has been continuously improved, making it an important technology in the nonwoven industry. It is favored by an increasing number of companies due to its simple process, short production time, and wide range of applications. Melt-blown nonwovens have also been applied in various fields, including filter materials, absorbers, and patterns. MBF is produced using the melt-blown method, which is a superfine fiber with a higher melting index (MI—amount of thermoplastic substances extruded within a specific time under defined conditions) by drawing a thin stream of the polymer melt, which is extruded from an orifice die through high-speed hot air [20]. The production process is detailed in Figure 1a.

The melt-blown fabric was selected as the modifier and chosen from the middle layer of the masks recycled from dustbin. The waste masks were recycled and sterilized using standard sterilization methods. First, we sprayed a 75% concentration of rubbing alcohol on both the front and back sides for the initial disinfection. Three to ten minutes later, the mask was cut, and the middle layer of melt-blown was taken out. Then, we carried out the second disinfection for a time of 0.5–2 min. The melt-blown was manually cut into pieces after the alcohol had completely evaporated. The size of melt-blown fabric taken from a waste mask was approximately 12 cm^2^. Previous tests [21] found that the MBF tended to mix and shear unevenly in the bitumen when the pieces were cut into 6 cm^2^ or 3 cm^2^. This is because the final modified bitumen was found to have agglomeration while being stirred with iron chopsticks. Based on the study [22], it was eventually cut into pieces measuring 1 cm^2^ (see Figure 1b). The microstructures of the MBF pieces observed by scanning electron microscopy (SEM) are shown in Figure 1c.

**Figure 2:**
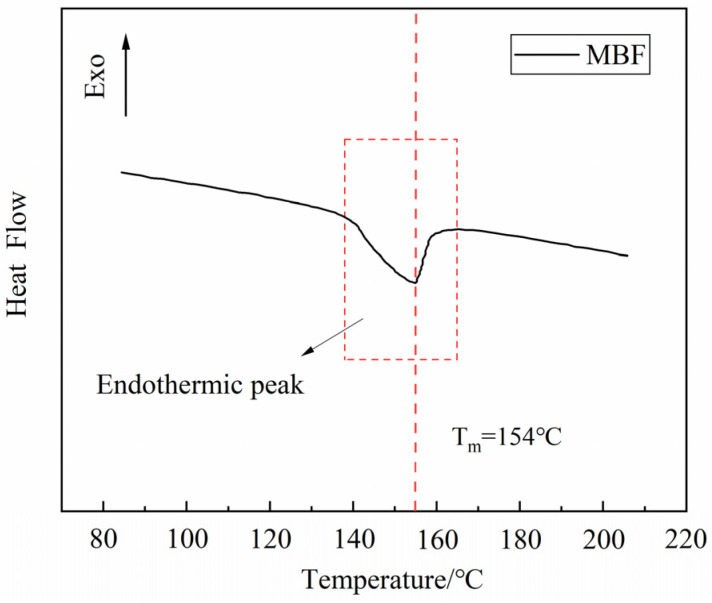
DSC results of MBF.

The melting point of MBF was measured using differential scanning calorimetry (DSC) in Figure 2. The physical parameters of MBF are provided in Table 3.

### 2.2. Methods

#### 2.2.1. Orthogonal Experimental Design (OED)

OED is a statistical method in mathematics that arranges and organizes experiments during the research process. It uses a set of normalized “orthogonal tables” to design experiments involving multiple factors, thereby reducing the number of tests to a certain extent [23]. It is one of the most commonly used experimental design methods due to its simplicity, effectiveness, and efficiency. There are many factors involved in the preparation process of MBF-modified bitumen. The orthogonal array (L_9_, 3^4^) was ultimately chosen to design the preparation process of MBF-modified bitumen. According to the orthogonal array, the specific experiment scheme is shown in Table 4.

#### 2.2.2. Preparation of Samples

**Figure 3:**
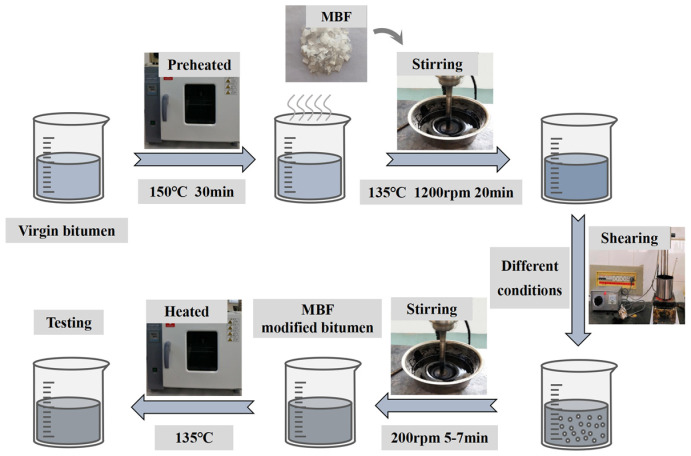
Preparation process of samples.

The virgin bitumen was heated in an oven at 150 °C until it reached a molten state. Then, pieces of MBF were gradually added, with concentrations of 1%, 3%, and 5% based on the percentage of bitumen. Then, they were stirred at 135 °C for 20 min to ensure uniform mixing. Then, the modifier was completely dissolved by using a shearing mixer under different conditions (3000–5000 rpm; 150–170 °C; 15–45 min). The MBF-modified bitumen was eventually obtained by stirring for 5–7 min to eliminate the bubbles produced by high-speed shear in the sample. It was heated at 135 °C for subsequent tests. The entire preparation process is schematically shown in Figure 3.

#### 2.2.3. Grey Relational Analysis (GRA)

GRA is a statistical analysis method that deals with multiple factors. It is used to solve the correlation problem in systems with incomplete information [24]. The results of the OED were analyzed using GRA, and a synthetic weighted score was calculated to explore the optimal preparation process for MBF-modified bitumen. The procedures are as follows:

(1) Set the reference sequence $X_{0}$ and the comparison sequence $X_{i}$ of the gray system:
  (1) $$X_{0}=\left[x_{0}\left(1\right),x_{0}\left(2\right),\;x_{0}\left(3\right)\ldots \ldots x_{0}\left(n\right)\right]$$
  (2) $$X_{i}=\left[x_{i}\left(1\right),x_{i}\left(2\right),\;x_{i}\left(3\right)\ldots \ldots x_{i}\left(n\right)\right]$$
  where: $X_{0}\left(i\right)$ is the ideal value in every evaluation index.
(2) Carry out the dimensionless calculation of data:
  (3) X*=Xi−XminXmax−Xmin
  (4) X*=Xmax−XiXmax−Xmin
(3) Calculate the correlation coefficient $\xi_{\left(Xi\right)}$:
  (5) $$\xi_{\left(Xi\right)}=\left(\Delta_{min}+\rho \Delta_{max}\right)/\left(\Delta_{ot}\left(t\right)+\rho \Delta_{max}\right)$$
  where $\Delta_{ot}\left(t\right)$ is the difference between the sequences, $\Delta_{ot}\left(t\right)=\left|x_{0}\left(t\right)-x_{i}\left(t\right)\right|$; $\Delta_{max}$ and $\Delta_{min}$ are the maximum and minimum values of the difference, respectively; ρ is the resolution factor, ρ∈ (0, 1), ρ= 0.5 in this research.
(4) Calculate the grey relational degree $R_{i}$:
  (6) $$R_{i}=\frac{1}{n}\sum_{i=1}^{n}\xi_{oi\left(t\right)}$$
  where: *n* is the number of data in the sequence.
(5) Calculate the synthetic weighted score:
  (7) $$Z_{i}=\sum_{j=1}^{5}b_{ij}\times \left(results\;of\;each\;index\right)$$
  where: $b_{ij}=\frac{R_{i}}{\Delta_{max}}$; j is the number of factors.

#### 2.2.4. Test Methods

Some physical property tests were performed according to the specification in Table 1. The penetration and softening point test was conducted using SYD-2801F and SYD-2806E, respectively. The ductility test was carried out at 10 °C by SYD-4508G tester with a tensile rate of 5 cm/min. The viscosity test was conducted at 135 °C by a Brookfield viscometer named NDJ-1F. All the instruments are produced by Shanghai Changji Geological Instrument Co., Ltd. (Shanghai, China).

The viscoelastic characteristics of the bitumen can be evaluated through dynamic shear rheological (DSR) test. It was conducted with an advanced rotary rheometer model MCR302 from the Anton Paar Company in Austria. The temperature sweep (TS) test was conducted according to ASTM D7175 [25], with a temperature range from 46 °C to 82 °C. To ensure rheological property tests were carried out within the linear viscoelasticity region of bitumen, all the binders underwent a strain sweep test.

**Figure 4:**
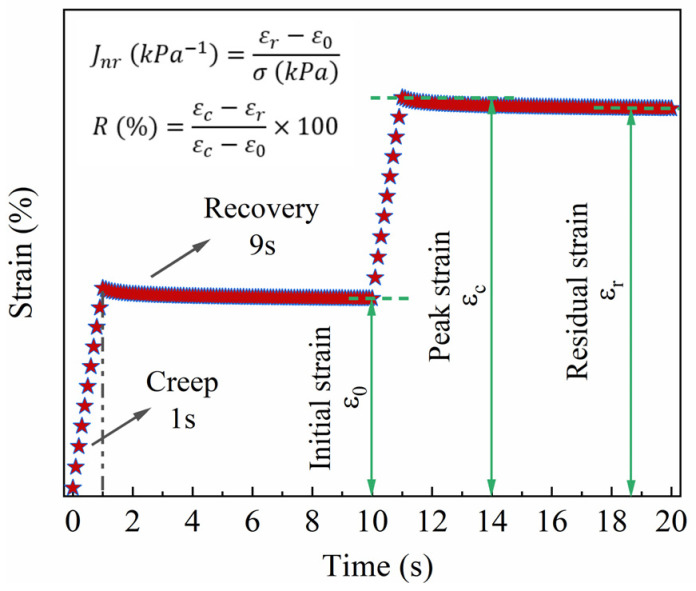
Test process and results of MSCR test.

The multiple stress creep and recovery (MSCR) test is applied to better reflect the non-linear viscoelastic performance of bitumen at high temperatures. According to AASHTO TP70 [26] (American Association of State Highway and Transportation Officials, MSCR test standard method), each test cycle consists of two stresses and two phases. The test process and two parameters, i.e., percent recovery (R) and non-recoverable compliance (Jnr), are shown in Figure 4. DSR test with a diameter of 25 mm is suitable for high-temperature tests above 40 °C. Most studies display that the R of bitumen starts to be negative at 0.1 kPa when the temperature is higher than 76 °C, manifesting that the viscous performance of virgin bitumen at higher temperatures is more apparent, while the elastic performance primarily vanishes. The changes in binder performance make it difficult to recover from deformation after loading. Correspondingly, the performance is opposite when the temperature is lower than 52 °C. Therefore, the temperature range is determined to be from 52 °C to 76 °C to make the test temperature of MSCR more accurate and save resources.

Bending beam rheological (BBR) test was applied to accurately assess the resistance to the low-temperature deformation of modified samples. It was conducted with a thermo-electric bending beam rheometer from the CANNON Instrument Company in America (State College, PA, USA). According to ASTM D6648 [27], a load of 980 ± 50 mN was consistently applied to the samples at temperatures of −12 °C, −18 °C, and −24 °C.

Fourier transform infrared spectroscopy (FTIR) test was conducted using Thermofisher’s Nicolet iS50 with a resolution of 0.09 cm^−1^. The spectral range was scanned from 4000 cm^−1^ to 400 cm^−1^. The fluorescence microscopy (FM) test was performed using an upright high-resolution fluorescence microscope named Zeiss Axio Imager A2 manufactured by Carl Zeiss IMT Co., Ltd. (Pudong, Shanghai, China).

**Figure 5:**
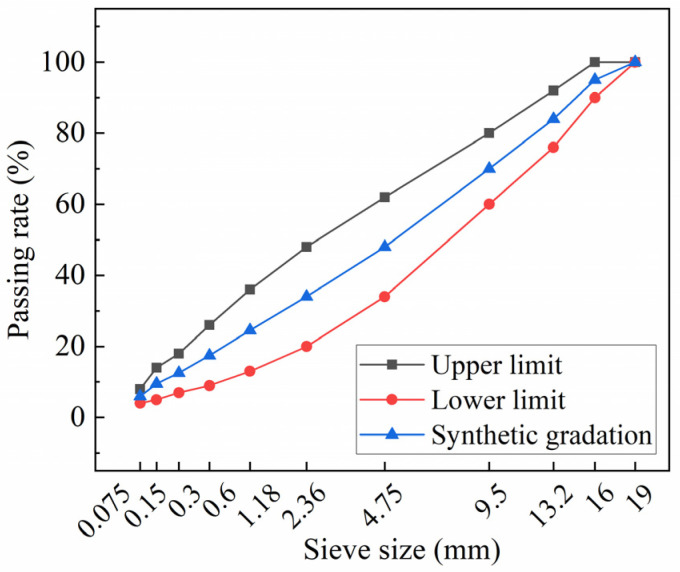
Aggregate gradation of AC-16.

According to JTG E20-2011 [28], the optimum binder content for all samples was found to be 4.7%. The AC-16, a type of dense-graded asphalt mixture, was selected for the test. The aggregate gradation, which represents the distribution of aggregate particles, is presented in Figure 5. The wheel tracking test was conducted to assess the resistance to the high-temperature deformation of asphalt mixture pavement bearing traffic loads. The beam bending test was performed to explore the performance of asphalt mixtures at low temperatures. The freeze–thaw indirect tensile strength test and water-immersed Marshall test were applied to illustrate the ability of asphalt mixtures to resist water damage where the tensile strength ratio (TSR) and residual Marshall stability ($MS_{0}$) were the final evaluation indicators. The equations can be obtained from [29].

#### 2.2.5. Radar Chart Evaluation Method (RCEM)

Comprehensive Evaluation (CE) refers to a method of evaluating a multi-index system using systematic and standardized evaluation methods. The radar chart analysis is a typical one in CE, which is intuitive and clear. However, it is difficult for the traditional RCEM to explore the ranking of comprehensive evaluation when there are numerous evaluation objects. In this paper, the advanced RCEM is performed to quantitatively explore the influence of MBF on the pavement performance of an asphalt mixture.

(1) Establish a matrix for the evaluating indicators:

Vector $X=\left\{x_{1}{,x}_{2},\cdots ,x_{n}\right\}$ and $Y=\left\{y_{1}{,y}_{2},\cdots ,y_{k}\right\}$ represents a set of objects to be evaluated and its indicators, respectively. Matrix $A={\left(a_{ij}\right)}_{n\times k}\left(i=1,2,\cdots ,n,\;j=1,2,\cdots ,k\right)$.

**Figure 6:**
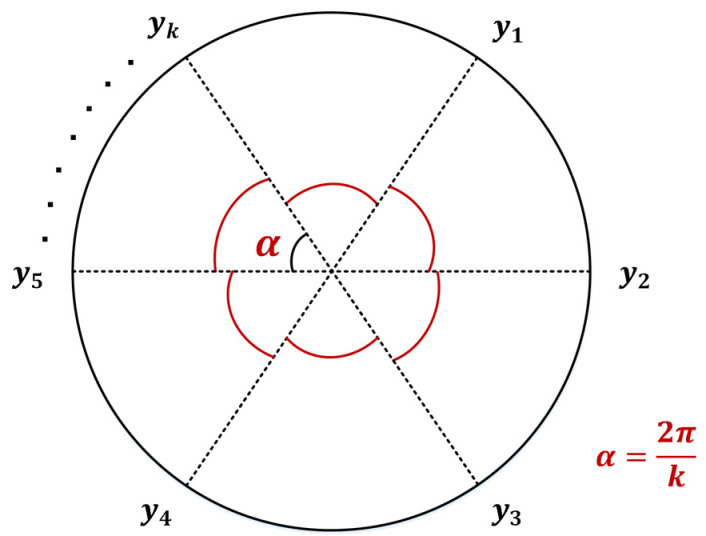
Sketch map of RCEM.

(2) Standardize the data in matrix A:
  (8) $$b_{ij}=\left(a_{ij}-E\left(y_{j}\right)\right)/\sigma \left(y_{j}\right)$$
  where $b_{ij}$ is the standardized data; $E\left(y_{j}\right)$ is the average value of indicator j, $E\left(y_{j}\right)=\frac{1}{k}\sum_{j=1}^{k}a_{ij}$; and $\sigma \left(y_{j}\right)$ is the standard deviation of indicator j, $\sigma \left(y_{j}\right)=\sqrt{\frac{1}{k}\sum_{j=1}^{k}{\left(a_{ij}-E\left(y_{j}\right)\right)}^{2}}$.
(3) Perform a non-linear transformation on every indicator:
  (9) $$r_{ij}=2/\pi \arctan\left(b_{ij}\right)+1$$
  where $r_{ij}$ is the indicator after non-linear transformation.
(4) Compute the characteristic vectors:
  (10) $$U_{i}=\left[A_{i},L_{i}\right]$$
  where $U_{i}$ is the evaluation vector of object i; $A_{i}$ and $L_{i}$ is the area and perimeter of the radar chart, respectively. The calculation formulas are as follows:
  (11) $$A_{i}=\sum_{j=1}^{k}1/k\pi {r^{2}}_{ij}$$
  (12) $$L_{i}=\sum_{j=1}^{k}2/k\pi r_{ij}$$
  (13) α=2π/k
  where k is the number of indicators; α is the central angle formed by adjacent indicators. The sketch map of RCEM is shown in Figure 6.
(5) Define the evaluation vector:
  (14) $$Z_{i}=\left[Z_{i1},Z_{i2}\right]$$
  where $Z_{i1}$ and $Z_{i2}$ is the relative area and perimeter of the evaluation object, respectively. The calculation formulas are as follows:
  (15) $$Z_{i1}=A_{i}/\max A_{i}$$
  (16) $$Z_{i2}=L_{i}/2\pi \sqrt{\frac{A_{i}}{\pi }}$$
(6) Compute the comprehensive evaluation results:
  (17) $$f\left(Z_{i1},Z_{i2}\right)=\sqrt{Z_{i1}\cdot Z_{i2}}$$

## 3. Results

### 3.1. OED and GRA

Table 5 presents the results of the OED and GRA. From Table 5, the modified bitumen prepared in the first group possessed the highest score. However, the ductility of the first group was 62.3 cm, which was far from the other eight groups. That is, the ductility was better than other groups obviously but the comprehensive performance was poor. This resulted in a high score that prevented it from being selected as the best preparation process. Settling for second best, the fifth group was selected. According to the standard design table, the optimum content of the MBF-modified bitumen was 3%, and the recommended shear time, rate, and temperature were 15 min, 4000 r/min, and 170 °C, respectively. 

**Figure 7:**
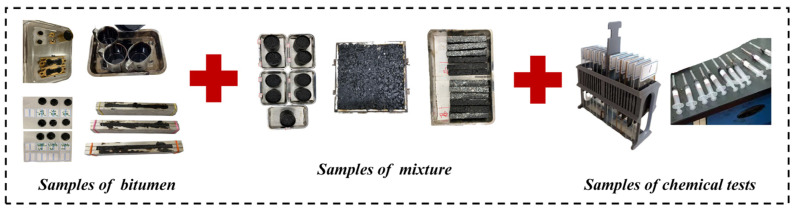
Samples prepared in the study.

To study the influence of MBF on the properties of bitumen and mixture, samples were prepared with varying concentrations of MBF (1%, 3%, and 5%) using the aforementioned preparation process. See Figure 7.

### 3.2. Properties of Bitumen

#### 3.2.1. Conventional Physical Properties

**Figure 8:**
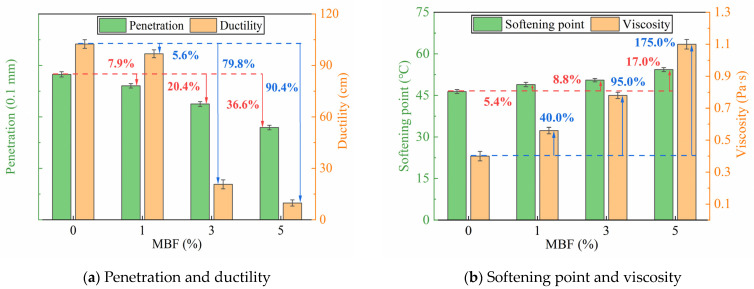
Results of physical property tests.

Figure 8 presents the results of the physical tests of the bitumen. As depicted in Figure 8, the penetration and ductility of MBF-modified bitumen decreased with higher MBF contents, while the softening point and viscosity exhibited a noticeable increase. This trend aligns with previous research findings [12]. The stiffness and high-temperature performance of bitumen can be characterized by penetration at 25 °C [30]. The penetration values decreased from 84.8 to 53.8, representing a 36.6% decline in the virgin bitumen. This decrease was accompanied by an increase in the MBF content from 0 to 5%. It was credited to the higher consistency of the MBF-modified bitumen, which in turn, would make the bitumen less sensitive to mechanical damage such as extrusion, collision, and impact. An obvious decrease in ductility can also be found, which resulted from the sharp increase in hardness. In contrast, the stiffening effect of MBF improved the softening point of the bitumen. It can be generalized that the softening point displayed a consistent enhancement; the increase ranges were 5.4%, 8.8%, and 17.0%, which showed that the trend of improvement strengthened with the increasing concentrations of MBF. The softening point is utilized to measure the stability of the bitumen at high temperatures [31]. The results illustrate that the high-temperature performance of the bitumen was improved by MBF and the improvement was more pronounced with higher concentrations of MBF. In brief, the modified bitumen demonstrated improved resistance to permanent deformation compared to the virgin bitumen.. Furthermore, the bitumen is supposed to be fluid enough to mix easily with aggregates. As shown in Figure 8b, MBF enhanced the viscosity of the bitumen. The viscosity continued to increase, growing by 175%, as the concentration of MBF increased from 0 to 5%. The maximum viscosity was 1.1 Pa·s, which meets the Superpave standard (less than 3 Pa·s at 135 °C). The results verified that the MBF-modified bitumen exhibited superior adhesion ability and adequate workability during construction. The added MBF was fibrous with some raised and striped textures on the surface. The rough fiber surface stabilized the bitumen, increasing its stiffness to a certain extent [32]. The following research and analysis on the blending mechanism in preparation also confirmed this phenomenon. Moreover, the fibrous MBF formed tiny reinforced networks in the bitumen by overlapping, thereby increasing the creep resistance. This was another reason that caused the increase in viscosity.

#### 3.2.2. Rheological Properties

**Figure 9:**
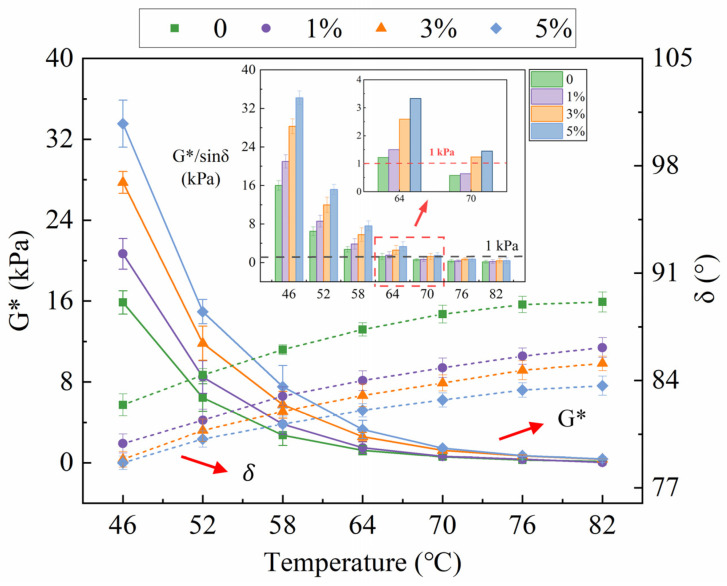
Results of TS test.

The results acquired from the TS test include the complex modulus (G*), phase angle (δ), and rutting factor (G*/sinδ). See Figure 9. G* is defined as the ratio of stress to strain subjected to dynamic load, which characterizes the ability to resist shear deformation [18]. From Figure 9, G* declined gradually with the increasing temperature, certifying that the ability to resist deformation gradually decreased. Moreover, the added MBF increased the G*. The virgin bitumen showed the lowest G* while the 5% MBF-modified bitumen performed the highest value, which was evident across the entire temperature range. The results of G* demonstrated that the added MBF could raise the capability of the bitumen to withstand permanent deformation at high temperatures.

The bitumen performs both elastic and viscous properties under normal service conditions. Elastic components occupy a dominant role in low-temperature performance. Conversely, this displays the performance of non-Newtonian viscous fluid at high temperatures, with the viscous components increasing [33]. The variation in the viscoelastic response of bitumen can be reflected by δ. Generally speaking, a lower δ value reveals higher elastic properties. Based on Figure 9, δ increased as temperature increased, suggesting a gradual decline in elasticity. Furthermore, the addition of MBF cut down the δ value relative to the control sample. This demonstrates that the elastic component in bitumen is enhanced, making it easier to recover from deformation. In short, the addition of MBF was favorable for improving the flexibility of asphalt pavement. A primary factor would result in the variation of G* and δ, in which MBF was swollen by the oily component to create two interlocked phases in the bitumen, causing the enhancement in hardness [34].

The rutting factor G*/sinδ is an important indicator for characterizing the rutting resistance of bitumen [35]. Generally, bitumen with a larger G*/sinδ is recognized to possess a better ability to resist rutting deformation. It is regarded to be durable to rutting deformation, as the value of original G*/sinδ is above 1 kPa [36]. As observed in Figure 9, the rutting factor for all samples decreased with the enhancement of temperature, indicating a reduction in their ability to resist rutting deformation. Additionally, the presence of MBF increased G*/sinδ, with 5% MBF-modified bitumen achieving the highest value. G*/sinδ of all samples met the requirements at 64 °C, but only 3% and 5% MBF-modified bitumen remained when the temperature rose to 70 °C. It is certified that the sample modified by MBF was more resistant to permanent deformation and had better high-temperature stability than the virgin bitumen. The chain length and molecular structure of bitumen and MBF vary widely [37]. The friction within the molecular structure of bitumen increased with the addition of MBF, resulting in an enhanced ability of bitumen to resist deformation. 

**Figure 10:**
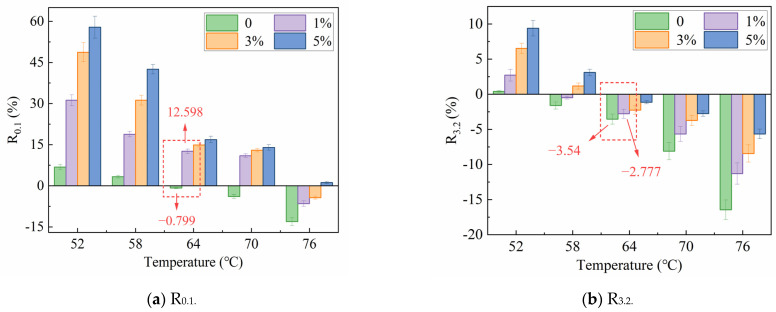

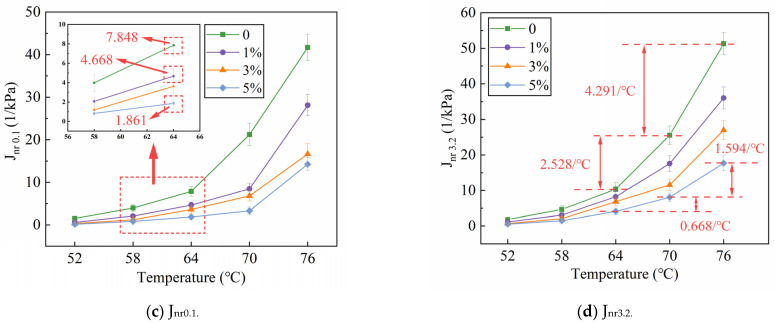
Results of MSCR test.

The two parameters of bitumen, i.e., non-recoverable compliance (Jnr) and percent recovery (R), were obtained in the MSCR test, as shown in Figure 10. The rutting deformation and elastic component of the binder can be evaluated by Jnr and R, respectively [38]. From Figure 10, it can be observed that the virgin bitumen exhibited the highest Jnr and the lowest R values, while the modified bitumen with 5% MBF showed the opposite trend. This indicates that the addition of MBF can enhance the resistance to rutting to some extent. Specifically, the modified bitumen with 5% MBF was the best. In addition, it declined gradually with the temperature enhancement, displaying that the elastic components in bitumen decreased, resulting in a reduction in the deformation recovery ability.

As shown in Figure 10, R_0.1_ was significantly higher than R_3.2_. This is consistent with the fact that rutting deformation is more likely to occur when the asphalt pavement bears a heavier traffic load. The increasing stress level reduced R from −0.799 to −3.54, showing a rate of change of 343.1%. The reduction in R under similar conditions with 1% MBF was from 12.598 to −2.777, displaying a rate of change of 122.0%. This was more than 1/3 of the virgin bitumen that manifested the enhanced reducing impact on the stress susceptibility by MBF. Similarly, with the incorporation of 1% MBF, Jnr decreased from 7.848 to 4.668 at 0.1 kPa. For 5% MBF, it decreased to 1.861, and there was a similar decrease in J_nr3.2_. The consistent decline in J_nr0.1_ and J_nr3.2_ reinforced the fact that the added MBF made the bitumen stiffer. Moreover, as the temperature rose from 64 °C to 76 °C, the growth rate of J_nr3.2_ of virgin bitumen changed from 2.528/°C to 4.291/°C, and the 5% MBF-modified bitumen varied from 0.668/°C to 1.594/°C, which occurred similarly at 0.1 kPa. This confirmed that MBF could reduce the temperature sensibility of bitumen.

**Figure 11:**
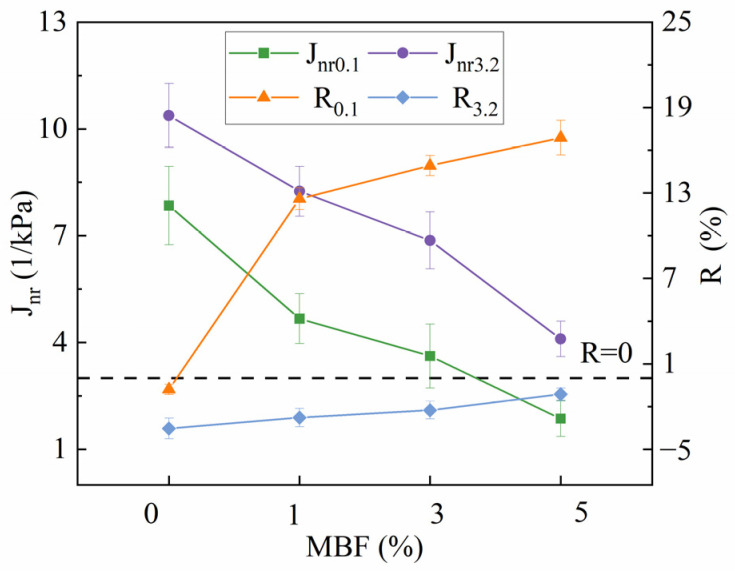
Changing of Jnr and R at 64 °C.

Taking the median temperature of 64 °C as the standard, the changing of J_nr_ and R with MBF is depicted in Figure 11. From Figure 11, the R value started to become negative at 0.1 kPa when the temperature was 64 °C, manifesting that the viscous behavior of the virgin bitumen at higher temperatures was more apparent, while the elastic performance primarily vanished. The changes in binder performance have made it difficult to recover from deformation after loading. Correspondingly, the R of the modified bitumen did not become negative until 76 °C, which was 12 °C higher than that of the virgin bitumen. This confirms again that MBF can improve the capability to resist the high-temperature deformation of bitumen. Moreover, there were differences in the change of R under two stress levels, which were reflected in a gradual increase in R_3.2_ and a rapid increase in R_0.1_. The changes in J_nr0.1_ and J_nr3.2_ were relatively stable, and the difference was imperceptible. The improvement of modified binders with the same content interval was uniform, which was aligned with the large deformation and small recovery of the plastic polymer-modified bitumen under creep load [39].

**Figure 12:**
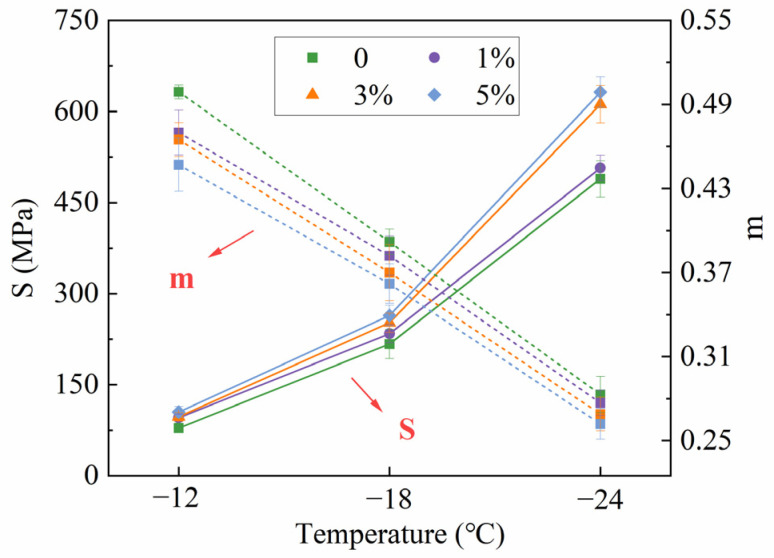
Results of BBR test.

The creep stiffness (S) and relaxation (m) shown in Figure 12 were recorded during the BBR test. Bitumen with a larger m and smaller S exhibited better performance at low temperatures. From Figure 12, MBF enhanced the S of bitumen, especially at the higher content of added MBF. In contrast, the m showed an opposite trend. The m is the slope of the stiffness curve, which indicates the ability to relax stresses [40]. Results demonstrated that MBF reduced the ability of stress relaxation and the low-temperature properties of bitumen, indicating a potential for low-temperature thermal cracking.

One of the important achievements of Strategic Highway Research Program (SHRP) is the Performance Grade (PG) system, which includes the evaluation of the high and low-temperature performance of bitumen. It is a comprehensive measurement of bitumen at different temperatures, reflecting the properties of bitumen comprehensively. The results of the PG grade are summarized in Table 6. It can be observed that the PG grade of bitumen was improved from PG 64-28 to PG 70-28 (where the maximum design temperature was improved from 64 °C to 70 °C) with 3% MBF. The design grade of high temperature has increased by one level, indicating that MBF can improve the high-temperature performance of bitumen. However, the PG grade with little MBF was the same as the virgin bitumen. That is, the improvement in high-temperature performance was indistinctive when little MBF was added, which was consistent with the results of the softening point. It can also be observed that there was no change in the low-temperature grade of PG with the addition of MBF, which illustrated that MBF has a relatively minor effect on the low-temperature rheological performance of binders.

For a median temperature of −18 °C, the limiting temperature difference (${∆T}_{C}$) was calculated to evaluate the degree of the hardening of binders. From Table 6, $T_{S=300\;\mathrm{MPa}}$ > $T_{m=0.3}$. This indicates that the critical failure temperature of binders is controlled by creep stiffness according to AASHTO M320. In addition, ${∆T}_{C}$ of modified bitumen increased compared to the virgin, indicating a decrease in the relaxation ability of the binders. This resulted in the formation of a gel-type structure, which led to the poor cracking resistance. But, the increase in ${∆T}_{C}$ was minimal, revealing that the addition of MBF had a slight negative impact on the low-temperature cracking resistance of modified binders.

#### 3.2.3. Mechanism of Blending

Based on the aforementioned research on the rheological properties at high and low temperatures, the study delves further into the blending mechanism of modification using waste masks.

**Figure 13:**
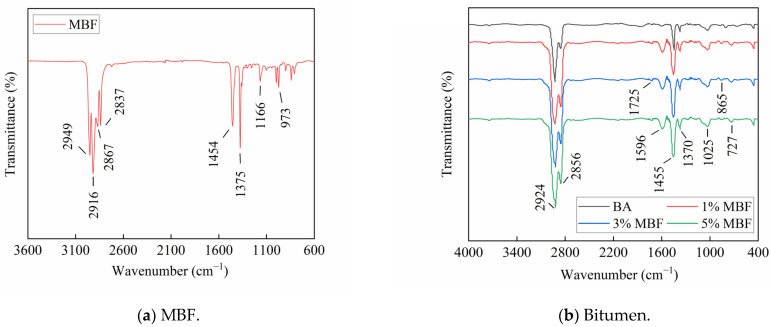
FTIR spectra of the samples.

From the spectra of MBF in Figure 13a, peaks occurring at 2949 cm^−1^, 2916 cm^−1^, 2867 cm^−1^, and 2837 cm^−1^ described the stretching vibration of the C–H bond. Meanwhile, the peaks at 1454 cm^−1^ and 1375 cm^−1^ were specific spectra containing CH_2_ deformation and symmetric CH_3_ deformation, which were the particular peaks of polypropylene (PP) [41]. The spectrum at the peak of 1166 cm^−1^ displayed the C–C bending, which is characteristic of the backbone of polypropylene (PP), followed by the wagging vibration of CH_3_ at 973 cm^−1^. The above analysis reveals that PP is the main component of MBF.

To investigate potential interactions during the modification process, FTIR spectra of bitumen were plotted in Figure 13b. Some strong peaks occurred at 2924 cm^−1^ and 2856 cm^−1^ because of the stretching vibration of the C–H bond [42]. These peaks above the four samples were almost identical, meaning that MBF rarely affected the chemical structure of bitumen. Some small peaks occurring near 1725 cm^−1^ and 1596 cm^−1^ were ascribed to the stretching vibration of C=O. The intensity of these peaks enhanced when the bitumen was aged during preparation [43]. Meanwhile, the absorption peaks between 1000–650 cm^−1^ were identified as being indicative of the presence of a benzene ring, suggesting substitution on the benzene ring. The spectra of all samples were similar to each other, but the peak intensities varied slightly. For example, the peak strength near 1600 cm^−1^ was stronger than that of the virgin bitumen, speculating that it had undergone slight aging during the preparation process. The intensity of the peaks was correlated with the temperature, solvent, and other conditions. It was difficult to control the strict consistency for each sample due to the limited experimental conditions. Therefore, the strength of some peaks at the same position on different curves varied. It can also be observed that the specific peaks of 1455 cm^−1^ and 1370 cm^−1^, which belong to MBF, remained in the spectrum. Above, the added MBF did not generate a new absorption peak of bitumen, suggesting a physical modification process rather than a chemical reaction. That is, the mechanism of blending in preparation was physical mixing.

**Figure 14:**
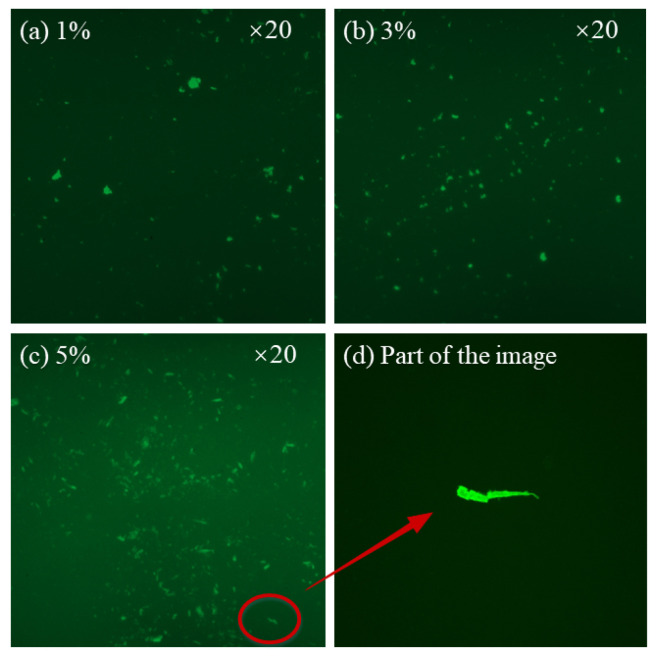
FM image of the bitumen.

To further understand the state of the modifier in bitumen after physical mixing and to assess the rationality of the preparation process, an FM analysis was carried out in Figure 14. It can be reported that there was no agglomeration, and the modifier was well-dispersed in the bitumen, revealing the success of the preparation process. The interface between MBF and bitumen was clear, and the morphology appeared to be relatively regular. This indicates that MBF finds it difficult to absorb enough oily phase and fully dissolve in the bitumen. From Figure 14c, the MBF particle seemed relatively slenderer in comparison with Figure 14a,b, resembling a fiber, as depicted in Figure 14d. It was observed that the modifier did not swell well during the preparation process when 5% MBF was added. Owing to the presence of two independent phases created by MBF and bitumen, stress concentration is prone to occur at the junction, making it susceptible to an easy fracture when stretched. That is, the ductility of modified bitumen was reduced. At the same time, the fibrous MBF absorbed the light components and increased the polymer-rich phase (PRP) in bitumen, resulting in a decrease in fluidity and plasticity. The decrease in ductility may also be caused by the fact that no effective bituminous membrane formed on the fiber surface, which made the bitumen brittle [44].

#### 3.2.4. Properties of Asphalt Mixture

**Figure 15:**
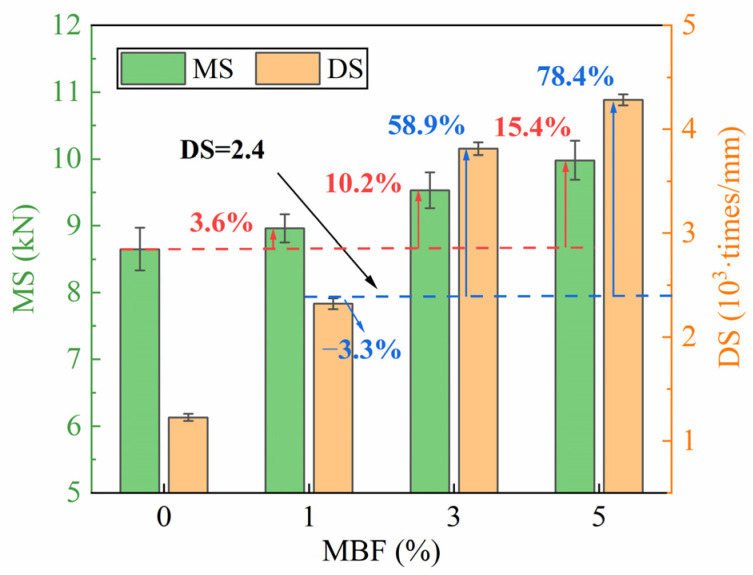
Results of high-temperature stability tests.

The results of the Marshall and wheel tracking tests can be observed in Figure 15. The force and deformation of the samples reveal the stiffness and flexibility of bitumen, which are correlated with the Marshall stability. A higher MS indicates that a relatively higher pressure is needed to break a sample of the bitumen. Figure 15 shows that MS gradually enhanced with increasing concentrations of MBF. The sample with 5% MBF had the best mechanical properties, which enhanced MS by 15.4% compared to the virgin bitumen. It was verified that MBF would make a great improvement to the mechanical property relative to the virgin. In addition, the modified asphalt mixture possessed a higher dynamic stability. It was −3.3%, 58.9%, and 78.4% higher than the specification (DS ≥ 2400 times/mm) when the MBF content was 1%, 3%, and 5%. Markedly, it did not meet the specifications when adding 1% MBF, verifying that the improvement in high-temperature performance was indistinctive with little MBF. The increasing Marshall stability and dynamic stability resulted from the modifier, which could resist greater deformation loads by enhancing the viscosity of the bitumen, tightening the combination of aggregates, and increasing the proportion of structural bitumen in the mixture through adsorption.

Table 7 shows the main indicators obtained from the beam bending test. It is visible that MBF enhanced the stiffness modulus of mixture and reduced the failure strain. The failure strain of the 1%, 3%, and 5% MBF-modified asphalt mixture was 6.8%, 10.5%, and 13.7% lower than the specification (ε ≥ 2800), respectively, suggesting that the more MBF was added, the lower the failure strain was. The ability to deform at low temperatures was affected by failure strain, where the lower the failure strain, the worse the flexibility at low temperatures and stress relaxation capability. To summarize, MBF weakens the ability of the mixture in low-temperature cracking. This is because MBF increased the creep stiffness modulus and reduced the stress relaxation ability of bitumen. At the same time, the molecular chain in a glassy state deteriorates the low-temperature cracking resistance of the MBF-modified asphalt mixture due to freezing.

**Figure 16:**
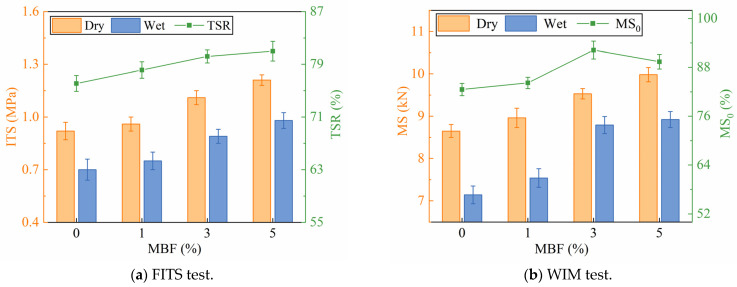
Results of moisture susceptibility tests.

The results of the freeze–thaw indirect tensile strength test (FITS) are presented in Figure 16a, where the TSR gradually increased as the MBF increased, indicating an improvement in moisture damage stability. However, TSR did not meet the specification (TSR ≥ 80%) when the MBF concentration was 1%, which indicated that the moisture damage stability of the mixture could be improved by a modifier, but the effect was limited to different proportions. Figure 16b shows the results of the water-immersed Marshall test (WIM), where the MS_0_ exhibited a similar trend to TSR. However, it can be noticed that the peak of MS_0_ occurred at 3% MBF. It is due to the higher residual stability when adding 3% MBF, which can be attributed to the increased viscosity that enhances its adhesion to the aggregate. Above, the resistance of asphalt mixtures to water damage could be improved by using a modifier. It could prevent water from entering the interface between the bitumen and the aggregate, which would delay the peeling off of bitumen from the surface of the aggregate.

### 3.3. RCEM Analysis

Although the properties of the MBF-modified asphalt mixture were evaluated, the effects of MBF have not been quantitatively explored at the same dosage. Therefore, the radar chart analysis was investigated. According to Equation (10), the characteristic vectors of matrix $A={\left(a_{ij}\right)}_{n\times k}={\left(a_{ij}\right)}_{4\times 5}$ were calculated as follows:

$$U_{1}=\left[A_{1},\;L_{1}\right]=\left[2.014,\;4.254\right];\;U_{2}=\left[A_{2},\;L_{2}\right]=\left[2.353,\;5.228\right]$$

$$U_{3}=\left[A_{3},\;L_{3}\right]=\left[5.299,\;7.959\right];\;U_{4}=\left[A_{4},\;L_{4}\right]=\left[5.298,\;7.751\right]$$

The evaluation vectors $Z_{i}=\left[Z_{i1},Z_{i2}\right]$ can be obtained as follows:

$$Z_{1}=\left[Z_{11},Z_{12}\right]=\left[0.380,0.846\right];\;Z_{2}=\left[Z_{21},Z_{22}\right]=\left[0.444,0.962\right]$$

$$Z_{3}=\left[Z_{31},Z_{32}\right]=\left[1.000,0.975\right];\;Z_{4}=\left[Z_{41},Z_{42}\right]=\left[0.999,0.950\right]$$

The comprehensive evaluation results $f\left(Z_{i1},Z_{i2}\right)=\sqrt{Z_{i1}\cdot Z_{i2}}$ were computed:

$$f_{1}=f\left(Z_{11},Z_{12}\right)=0.567;\;f_{2}=f\left(Z_{21},Z_{22}\right)=0.653$$

$$f_{3}=f\left(Z_{31},Z_{32}\right)=0.988;\;f_{4}=f\left(Z_{41},Z_{42}\right)=0.975$$

**Figure 17:**
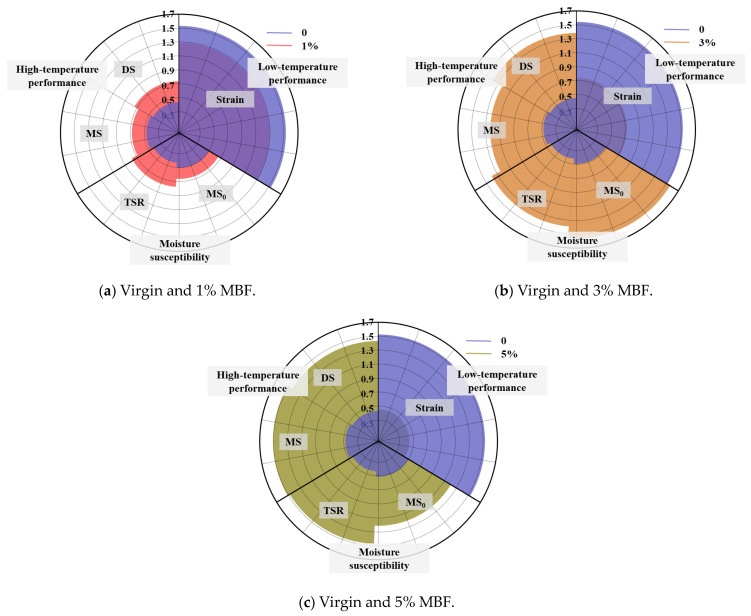
Radar charts of RCEM results.

In a word, the final evaluation results of MBF for enhancing the properties of the mixture indicated the following order: $f_{3}>f_{4}>f_{2}>f_{1}$. Markedly, the impact of 3% MBF was the most significant, followed by 5% MBF and 1% MBF. This was consistent with the OED and GRA results, in which the modified binder with 3% MBF possessed the best performance. The differences in the improvement of MBF properties are shown in Figure 17. This can provide some appropriate approaches for preparing the MBF-modified asphalt mixture with improved performance.

## 4. Conclusions

The utilization of the melt-blown fabric of waste masks as a modifier material to acquire the MBF-modified bitumen was studied in this research. The preparation process and properties of the MBF-modified bitumen were investigated. The blending mechanism during preparation and the homogeneity of the bitumen were analyzed. Meanwhile, the performance of the asphalt pavement mixture was studied, and a radar chart analysis was performed to quantitatively assess the effects of the MBF mixture.

The recommended preparation process of the shear time, shear rate, and shear temperature was 15 min, 4000 r/min, and 170 °C, respectively. The stiffness of bitumen was enhanced by a melt-blown fabric with decreased ductility, and the temperature susceptibility was significantly reduced.

The addition of the melt-blown fabric improved the capability of bitumen to resist high-temperature deformation. However, the ability of stress relaxation was reduced at low temperatures, indicating that the bitumen had the potential for thermal cracking.

The modification mainly involved physical mixing, with no new absorption peak generated. There was no agglomeration of modifiers and no network structure formed in the modified bitumen.

The mechanical properties of the asphalt mixture were improved by the melt-blown fabric, resulting in an increased resistance against permanent deformation and water damage, as well as a decreased low-temperature anti-cracking performance.

The comprehensive assessment results of MBF to improve the properties of the mixture were in the following order: $f_{3}>f_{4}>f_{2}>f_{1}$, where the impact of 3% MBF was the most significant, followed by 5% MBF and 1% MBF.

In summary, MBF-modified bitumen performs well at high temperatures, and the mixture exhibits a better pavement performance compared to the virgin asphalt mixture. It is recommended that 3% is the optimum content of bitumen modified by the melt-blown fabric of waste masks. The success of this research will encourage the application of the melt-blown fabric of waste masks in road construction and improve the environmental friendliness of the bitumen material.

## Figures and Tables

**Table 1:** Physical properties of virgin bitumen.

Physical Properties	Results	Technical Requirements	Test Methods
Penetration (25 °C, 0.1 mm)	84.8	80–100	ASTM D5 [13]
Ductility (10 °C, 5 cm/min, cm)	102.5	>20	ASTM D113 [14]
Softening point (°C)	46.4	>45	ASTM D36 [15]
Viscosity (135 °C, Pa·s)	0.4	<3	ASTM D4402 [16]

**Table 2:** Parameters of the original polymer [17,18].

Parameters	Information
Chemical Formula	(C_3_H_6_)_n_
Appearance	Translucent solid
Density (g/cm^3^)	0.89–0.91
Melting point (°C)	164–170

**Table 3:** Physical parameters of MBF.

MI	Melting Point	Diameter
(150 ± 10) g/min	150–160 °C	0.5–10 μm

**Table 4:** Experiment scheme.

Numbers	MBF (%)	Shear Rate (rpm)	Shear Temperature (°C)	Shear Time (min)
1	1	3000	150	15
2	1	4000	160	30
3	1	5000	170	45
4	3	3000	160	45
5	3	4000	170	15
6	3	5000	150	30
7	5	3000	170	30
8	5	4000	150	45
9	5	5000	160	15

**Table 5:** Results of OED and GRA.

Projects	$\mathrm{Pen}.\;(\xi_{01}$)	$\mathrm{SP}.\;(\xi_{02}$)	$\mathrm{Duc}.\;(\xi_{03}$)	$\mathrm{Vis}.\;(\xi_{04}$)	Score (10^2^)
X_0_	30.7	58.3	62.3	1.04	-
X_1_	69.5 (0.33)	47.2 (0.33)	62.3 (1.00)	0.60 (0.33)	4390.24
X_2_	51.9 (0.48)	50.6 (0.42)	13.5 (0.37)	0.62 (0.34)	3026.83
X_3_	30.7 (1.00)	58.3 (1.00)	4.8 (0.33)	0.63 (0.35)	2469.15
X_4_	42.0 (0.63)	53.3 (0.53)	9.4 (0.35)	0.93 (0.67)	2750.62
X_5_	55.2 (0.44)	50.6 (0.42)	15.2 (0.38)	0.80 (0.48)	3156.38
X_6_	45.6 (0.57)	53.8 (0.55)	8.2 (0.35)	0.63 (0.35)	2831.16
X_7_	44.1 (0.59)	54.5 (0.59)	7.7 (0.34)	1.04 (1.00)	2808.94
X_8_	39.4 (0.69)	55.4 (0.66)	7.3 (0.34)	1.02 (0.92)	2694.07
X_9_	45.5 (0.57)	52.6 (0.49)	9.9 (0.35)	0.94 (0.69)	2839.26
R_i_	0.589	0.554	0.423	0.570	-

**Table 6:** PG grade and $\Delta T_{C}$ of the bitumen.

MBF (%)	PG	$T_{S=300\;\mathrm{MPa}}$	$T_{m=0.3}$	${∆T}_{C}$
0	PG 64-28	−19.83	−23.06	3.23
1%	PG 64-28	−19.45	−22.69	3.24
3%	PG 70-28	−18.80	−22.16	3.36
5%	PG 70-28	−18.59	−21.71	3.12

**Table 7:** Results of the beam bending test.

MBF (%)	Tensile Strength (MPa)	Failure Strain (με)	Stiffness Modulus (MPa)
0	7.75	2673.35	2898.98
1%	7.98	2610.02	3057.45
3%	8.31	2507.37	3314.23
5%	8.72	2432.27	3585.13

## Data Availability

Data are contained within the article.
